# *TRAPPC9*-Related Intellectual Developmental Disorder: A Systematic Review and a Novel Case of a Complex Structural Variant

**DOI:** 10.3390/genes17060658

**Published:** 2026-06-03

**Authors:** Marta Calvo, Giuseppe Reynolds, Maria Luca, Eleonora Di Gregorio, Simona Cardaropoli, Eliana Salvo, Ilaria Carelli, Federico Rondot, Stefania Massuras, Diana Carli, Roberta Marinoni, Maria Clara Bonaglia, Alessandro Mussa

**Affiliations:** 1 Department of Public Health and Pediatrics, University of Turin, 10126 Turin, Italy; marta.calvo@unito.it (M.C.); giuseppe.reynolds@unito.it (G.R.); simona.cardaropoli@unito.it (S.C.); federico.rondot@unito.it (F.R.); 2 Postgraduate School of Pediatrics, Department of Public Health and Pediatrics, University of Turin, 10126 Turin, Italy; 3 Pediatric Clinical Genetics, Regina Margherita Children’s Hospital, 10126 Turin, Italy; maria.luca@unito.it (M.L.); stefania.massuras@unito.it (S.M.); diana.carli@unito.it (D.C.); 4 Medical Genetics Unit, Città della Salute e della Scienza University Hospital, 10126 Turin, Italy; edigregorio@cittadellasalute.to.it (E.D.G.); rmarinoni@cittadellasalute.to.it (R.M.); 5 Cytogenetics Laboratory, Scientific Institute, IRCCS Eugenio Medea, 23849 Lecco, Italy; eliana.salvo@lanostrafamiglia.it (E.S.); mariaclara.bonaglia@lanostrafamiglia.it (M.C.B.); 6 Department of Medical Sciences, University of Turin, 10126 Turin, Italy; ilaria.carelli@unito.it

**Keywords:** TRAPPC9, MRT13, NF-κB, intellectual disability, neurodevelopmental disorder, microcephaly, optical genome mapping

## Abstract

**Background**: Autosomal recessive intellectual developmental disorder-13 (MRT13; OMIM #613192) is a rare neurodevelopmental disorder caused by pathogenic variants in *TRAPPC9*. Most reported variants are single-nucleotide variants (SNVs), small insertions/deletions, or copy number variants (CNVs), whereas complex structural variants (SVs) remain poorly characterized. **Objectives**: This study sought to review the clinical and molecular spectrum of *TRAPPC9*-related disorder, harmonize reported variants, explore genotype–phenotype correlations, and expand the mutational spectrum by reporting a novel patient with a cryptic SV. **Methods:** We report a novel patient whose diagnostic workup included array-CGH, whole-exome sequencing, karyotyping, and optical genome mapping. Additionally, a systematic literature search was primarily conducted in PubMed/MEDLINE from 2009 to January 2026, with Embase, Web of Science, Google Scholar, Orphanet, OMIM, and ClinVar used as supplementary sources. Patients carrying pathogenic/likely pathogenic *TRAPPC9* variants were included. Clinical and molecular data were extracted and descriptively summarized. Genotype–phenotype correlations were explored. Reported variants were re-annotated using MANE Select reference transcripts. **Results**: The reported patient showed biallelic *TRAPPC9* disruption due to two independently inherited structural variants: a maternal ~35 kb intragenic deletion involving exons 10–12, identified by 400K array-CGH, and a paternal balanced translocation t(4;8) disrupting *TRAPPC9* within intron 8, characterized by trio-OGM and paired-end whole-genome sequencing (PE-WGS). Thirty-one studies reporting 75 previously published patients were included in the literature review; together with the novel patient described here, the final cohort comprised 76 patients. Intellectual disability was present in 100% of cases, followed by brain MRI abnormalities (95.9%), microcephaly (82.3%), motor delay (71.4%), dysmorphic features (69.8%), obesity (52.8%), behavioral abnormalities/autism spectrum disorder (49.2%/43.8%), and epilepsy (15.9%). Most patients (84.2%) harbored homozygous variants. Thirty-two distinct sequence variants were identified, predominantly loss-of-function. CNVs were identified in 13.2% of patients. No genotype–phenotype correlations were identified. **Conclusions**: The systematic review provides an updated and harmonized overview of the clinical and molecular spectrum of *TRAPPC9*-related disorder, supporting the presence of a recognizable phenotype and confirming the predominance of loss-of-function variants. Our case further highlights the contribution of cryptic structural variants to the mutational spectrum of *TRAPPC9* and the diagnostic value of advanced genomic approaches.

## 1. Introduction

Trafficking protein particle complex subunit 9 (TRAPPC9; OMIM #611966) is a component of the trafficking protein particle II (TRAPPII) complex [1], which is involved in membrane tethering of coated vesicles during intra-Golgi and endosome-to-Golgi trafficking. TRAPPC9 has also been implicated in ER-to-Golgi transport, broader intracellular vesicular trafficking, and regulation of NF-κB signaling pathways through its interaction with IKKβ and NF-κB-inducing kinase (NIK) [2]. Experimental models suggest that the TRAPPII complex regulates axonal and dendritic elongation and branching, and that *Trappc9* deficiency may impair these processes, potentially contributing to neurodevelopmental features such as microcephaly and intellectual disability [3].

The gene is located at chromosome 8q24.3 and comprises 23 exons; its MANE Select transcript, NM_001160372, encodes the canonical TRAPPC9 protein isoform. Pathogenic variants in *TRAPPC9* have been associated with autosomal recessive intellectual disability type 13 (MRT13; OMIM #613192) [4]. Individuals with MRT13 typically present with moderate to severe intellectual disability, postnatal microcephaly, and structural brain abnormalities [5]. Consistent with autosomal recessive inheritance, *TRAPPC9*-related intellectual developmental disorder has been predominantly described in consanguineous families [6,7,8]. Most pathogenic variants are single-nucleotide variants or small insertions/deletions, followed by copy number variants (CNVs), whereas the contribution of complex structural variants (SVs) remains poorly characterized.

To date, a growing number of patients with *TRAPPC9*-related intellectual developmental disorder have been reported, contributing to the definition of its clinical and molecular spectrum. However, the full extent of the associated phenotypic variability, the distribution of pathogenic variants across the gene, and genotype–phenotype correlations are far from being well-defined. Here, we report a novel patient harboring a compound heterozygosity for two distinct SVs, a paternally inherited balanced translocation t(4;8) and a maternally inherited intragenic deletion in *TRAPPC9*, further expanding the molecular and clinical spectrum of this condition. Furthermore, we undertook a systematic review aiming to: (i) comprehensively delineate the clinical phenotypic spectrum associated with *TRAPPC9*-related intellectual developmental disorder through individual-level analysis of all reported patients; (ii) systematically assess and harmonize the spectrum of reported *TRAPPC9* variants; and (iii) investigate potential genotype–phenotype correlations.

## 2. Materials and Methods

### 2.1. Case Report

For our case, data were collected prospectively as part of routine clinical care. A 60K array-CGH platform, conventional karyotyping and whole exome sequencing (WES) were performed as part of the standard diagnostic workflow. Subsequent molecular characterization was conducted with a 400K-resolution array-CGH (Agilent Technologies, Santa Clara, CA, USA) and Optical Genome Mapping (OGM, Bionano Genomics, Saphyr, San Diego, CA, USA). Written informed consent was obtained from the patient’s parents for genetic investigations and publication of clinical data.

### 2.2. Optical Genome Mapping (OGM) in the Trio

Ultra-high molecular weight gDNA (>150 kb) was extracted from the peripheral blood (EDTA) of the proband and his parents using the SP Blood & Cell Culture DNA Isolation Kit (Bionano Genomics, San Diego, CA, USA). The gDNA was labeled following the manufacturer’s instructions with the Bionano Prep Direct Label and Stain (DLS) protocol, and the data were analyzed on a Saphyr instrument (Bionano Genomics). A minimum of 500 Gb of data was collected. Bionano Solve v3.8.2 was used for de novo genome assembly at 80× coverage, trio analysis, variant calling, and annotation with default settings. Annotated variants were filtered for rare events (≤1% in the OGM control database), as described previously [9].

### 2.3. Pair-End Whole-Genome (PE-WGS) and Breakpoint Junctions Analysis

Genomic DNA was extracted from the proband’s blood with standard procedures and sequenced using an Illumina HiSeq 2000 platform (Illumina, San Diego, CA, USA) with a 30 × PCR-free PE-WGS protocol (Illumina, San Diego, CA, USA). Reads were mapped to the human reference genome GRCh38/hg38 using BWA [10]. Structural variants (SVs) were visualized and manually checked in the Integrative Genomics Viewer (IGV) genome browser.

### 2.4. Literature Review

This systematic review was conducted and reported according to the PRISMA 2020 guidelines. The review was not prospectively registered, and no separate review protocol was prepared.

A systematic literature search was primarily conducted in PubMed/MEDLINE, covering the period from 2009—the year of the first description of MRT13—through January 2026. The search strategy combined the terms (“TRAPPC9” OR “NIBP” OR “MRT13”) AND (“intellectual disability” OR “neurodevelopmental disorder”). Embase, Web of Science, and Google Scholar were subsequently searched as supplementary sources to identify additional relevant records not retrieved through PubMed/MEDLINE. Reference lists of included articles were also manually screened for additional studies.

Only articles published in English were considered. All retrieved records were manually compiled in a spreadsheet, and duplicates were identified and removed. Titles and abstracts were screened to identify potentially eligible studies, followed by full-text assessment according to predefined inclusion and exclusion criteria. Studies reporting patients of any age carrying pathogenic or likely pathogenic germline variants in *TRAPPC9* were included. Case reports, case series, cohort studies, and short communications were considered eligible. Functional or preclinical studies, somatic/oncologic studies, non-human studies, studies focused on other genes, and studies unrelated to *TRAPPC9*-related neurodevelopmental disorder were excluded. Reports with unavailable full text or insufficient clinical or molecular data were excluded.

In addition, ClinVar and DECIPHER were queried in May 2026 to provide a complementary overview of publicly submitted *TRAPPC9* variants. Database-derived variants were summarized separately from the literature-based patient cohort, as database entries often lack detailed individual-level clinical information, segregation data, and phenotypic annotation required for harmonized genotype–phenotype analysis.

Data were extracted at the individual patient level, as recommended for systematic reviews of ultra-rare disorders [11]. Extracted variables included demographic, genetic, perinatal, clinical, and neuroimaging data. Data extraction was performed by one reviewer and independently verified by a second reviewer.

Due to the predominance of case reports and small case series, formal risk-of-bias and certainty assessments were not performed. A quantitative meta-analysis was not feasible because of the rarity of the condition, limited sample size, and clinical and molecular heterogeneity; therefore, results were synthesized descriptively.

### 2.5. Variant Re-Evaluation and Re-Annotation

All reported *TRAPPC9* variants were annotated according to the Human Genome Variation Society (HGVS) nomenclature using the MANE Select reference transcript (NM_001160372), including, when necessary, conversion from alternative or outdated transcripts. This approach ensured uniformity in variant description across studies, enabled more reliable comparison between reported cases, and supported a more reliable assessment of genotype–phenotype correlations. Variants were interpreted according to the guidelines of the American College of Medical Genetics and Genomics (ACMG) [12].

### 2.6. Statistical Tests

Frequencies and percentages were calculated for categorical variables, and descriptive statistics (median and range) for continuous variables, considering only patients with available data for each parameter. Genotype–phenotype correlations were explored by stratifying clinical features according to variant type. Differences between categorical variables were assessed using Fisher’s exact test (two-tailed). A *p*-value < 0.05 was considered statistically significant.

## 3. Results

### 3.1. Our Case

**Figure 1:**
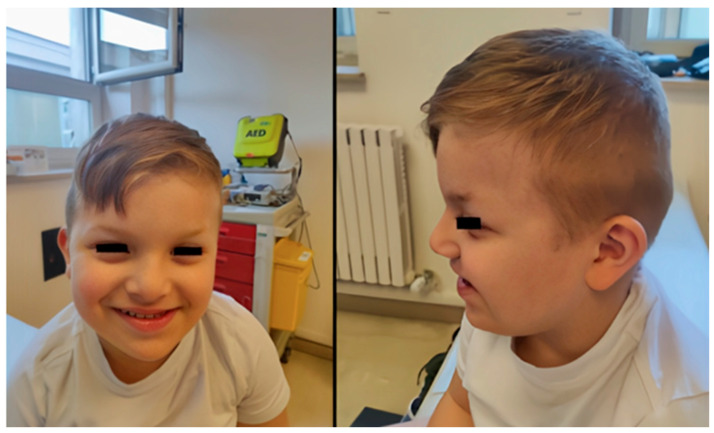
Frontal and lateral views showing dysmorphic features of our patient, including turricephalic skull shape, epicanthal folds, low-set ears, and lateral sparseness of the eyebrows. Photos obtained with written parental consent.

The proband, a male of Italian origin, presented with cerebral palsy, initially interpreted as secondary to a cryptic hypoxic–ischemic injury during pregnancy, global developmental delay, and moderate intellectual disability. Pregnancy was complicated by threatened preterm labor in the third trimester. He was born at term by spontaneous delivery (Apgar score 9/9), with appropriate-for-gestational-age growth parameters. Neonatal examination showed mild motor immaturity with transient tremors that resolved spontaneously. Independent walking was achieved after 24 months. Language development began at approximately 12 months but remained markedly delayed, with limited vocabulary at the last evaluation at 6 years of age. Cognitive assessment performed at 5 years using the WPPSI showed a full-scale intelligence quotient (FSIQ) of 50, consistent with moderate intellectual disability; detailed subscale scores were not available. Physical examination at 6 years of age revealed a height of 126 cm (+1.2 SDS) and weight of 55 kg (+3.86 SDS), with normocephalic head circumference. Dysmorphic features included turricephalic skull shape, epicanthal folds, low-set ears, and lateral sparseness of the eyebrows (Figure 1). Brain MRI performed at the age of 3 years demonstrated peritrigonal hyperintensities within the corona radiata, interpreted as sequelae of previous hypoxic–ischemic injury. EEG revealed bilateral frontocentral paroxysmal activity at sleep onset. Metabolic investigations, including newborn screening, urinary organic acids, and plasma acylcarnitines, yielded normal results. Audiological and ophthalmological evaluations, as well as abdominal ultrasound, were unremarkable.

Both parents were healthy, with no personal history of neurodevelopmental disorders. The proband’s younger sister presented with failure to thrive and mild developmental delay. Array-CGH analysis identified a terminal deletion involving 4q34.3–q35.2 (~8.7 Mb) together with a duplication of 8q24.3 (~5 Mb). Subsequent karyotype revealed an unbalanced translocation between chromosomes 4q and 8q, inherited from a reciprocal balanced translocation t(4;8)(q34.3;q24.3) in the father. Maternal karyotype was normal.

The first-tier 60K-array-CGH of the proband and the trio-based WES did not reveal causative CNVs or SNVs; a paternally inherited pathogenic heterozygous *ALG1* variant (c.1187+1G>A) was detected, consistent with carrier status for the autosomal recessive congenital disorder of glycosylation, which was unrelated to the proband’s phenotype. The 400K-resolution array-CGH subsequently detected a 31–39 kb microdeletion at 8q24.3 involving *TRAPPC9* (intron 9 to exon 12), below the detection limit of the 60K and 180K platforms. Provided the phenotypic concordance with autosomal recessive intellectual disability type 13 and the paternal translocation involving the chromosomal region of *TRAPPC9,* a pathogenic rearrangement affecting the paternal *TRAPPC9* allele was hypothesized and explored through OGM.

**Figure 2:**
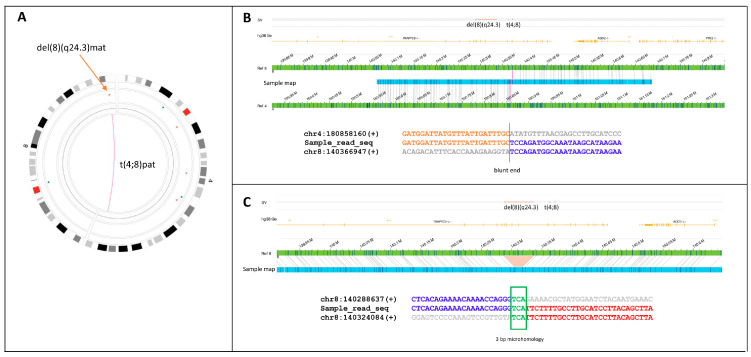
Optical Genome Mapping. (**A**) Circos plot: The central magenta lines connecting chromosomes 4 and 8 indicate a paternal reciprocal translocation. The orange dot (arrow) indicates the maternal 8q24.3 deletion. In both panels (**B**,**C**), the SV_track (**upper**) shows SV_calls for the t(4;8) and the 8q24.3 deletion. The blue bar represents the patient sample analyzed. The green bar corresponds to the reference genome for chromosomes 4 and 8. (**B**) Translocation t(4;8)(q34.3;q24.3) interrupting *TRAPPC9* within intron-8 (NM_001160372.4): one segment of the sample map aligns with chromosome 8, and another aligns with chromosome 4. The vertical pink line denotes the translocation breakpoints. PE-WGS confirmed the translocation breakpoint junction sequence by blunt-end repair (**bottom**). (**C**) Genome map showing the deletion involving exons 8–12 of *TRAPPC9* (NM_001160372.4): the red triangle denotes the ~35 kb loss. PE-WGS confirmed the deletion breakpoint junction sequence. Microhomology of 3 bps (TCA) between the sequences of chromosomes 8 and 4 is highlighted in green (**bottom**).

The trio-OGM analysis, combined with Paired-End Whole-Genome Sequencing (PE-WGS), refined the t(4;8) breakpoints, revealing disruption of *TRAPPC9* (NM_001160372.4) at 8q24.3 within intron 8, and confirmed a heterozygous ~35 kb deletion involving exons 10–12 (Figure 2). The translocation and deletion were inherited from the father and mother, respectively, resulting in compound heterozygosity. These findings led to a final diagnosis of *TRAPPC9*-related intellectual developmental disorder in the proband, resulting from biallelic disruption of *TRAPPC9* by two independently inherited pathogenic structural variants.

### 3.2. Systematic Review

The systematic literature search identified 75 patients with pathogenic or likely pathogenic *TRAPPC9* variants reported across 31 publications (Table S1) [4,5,6,7,8,13,14,15,16,17,18,19,20,21,22,23,24,25,26,27,28,29,30,31,32,33,34,35,36,37,38]. The study selection process is detailed in the PRISMA 2020 flow diagram (Figure S1). After inclusion of our newly described case, the final cohort comprised 76 patients included in the analysis.

#### 3.2.1. Clinical Characterization

Among the patients included in the literature review, sex was available for 75 individuals, with a slight female predominance (41/75, 54.7%). Ethnicity was reported for 72 patients, with most individuals originating from the Middle Eastern and North African (MENA) regions (34/72, 47.2%) and South Asia (19/72, 26.3%), followed by Caucasian populations (13/72, 18.1%), whereas other ethnic groups were underrepresented. Relatives’ consanguinity was documented in 58/71 patients (81.7%).

Clinical data were available for a variable number of patients depending on the feature analyzed (Table 1).

Intellectual disability was consistently reported in all evaluated individuals, and was classified as severe in the majority of cases (35/48, 66.7%). Brain MRI abnormalities were detected in nearly all evaluated patients, with white matter abnormalities and thinning of the corpus callosum being the most frequent findings. Additional neuroimaging features included cerebellar hypoplasia and reduced cerebral volume. Postnatal microcephaly and motor delay were also consistently observed throughout the cohort.

Dysmorphic features were frequently reported and, although heterogeneous, showed recurrent patterns. The most frequently reported craniofacial features included a round face, often associated with brachycephaly and a low anterior hairline. Common periorbital findings comprised synophrys, epicanthal folds, and hypertelorism, whereas a broad or prominent nasal bridge was consistently observed. Orofacial features included a short or smooth philtrum and a thin upper lip, and ear anomalies—particularly low-set or prominent auricles—were also frequently noted.

Obesity emerged as a common manifestation. Behavioral abnormalities were frequently described, most commonly including aggressive behavior, self-injurious tendencies, and hyperactivity. Among neurobehavioral manifestations, autism spectrum disorder was relatively frequent, whereas sleep disturbances, particularly frequent nocturnal awakenings, were reported less consistently. Epilepsy appeared to represent a less common feature within the cohort.

#### 3.2.2. Molecular Findings

Among the 76 patients included in the cohort, 64 (84.2%) harbored a homozygous variant, whereas 12 (15.8%) harbored compound heterozygous variants. No de novo variants were identified.

A total of 72 patients (94.7%) carried at least one sequence variant (SNV or small intragenic deletion/duplication). Among these, 61 were homozygous (84.7%), while 11 (15.3%) were compound heterozygous, including six cases with a sequence variant in combination with a CNV and five cases with two sequence variants.

Overall, 32 distinct SNVs were identified (Table 2). Of these, 20 (62.5%) were predicted loss-of-function, whereas 12 (37.5%) were missense variants. The most recurrent variant was the c.1129C>T, p.(Arg377Ter), reported in 16 patients (16/76, 21%), always in a homozygous state.

Overall, 10 patients (13.2%) harbored CNVs, including seven distinct deletions and two duplications, three of which were in a homozygous state (Table 3). Notably, our patient represents the only case with biallelic *TRAPPC9* disruption resulting from the combination of a CNV and a balanced translocation.

Genotype–phenotype comparisons were performed to assess potential differences in clinical features according to variant type. Patients harboring copy number variants were compared with those carrying sequence variants, and no statistically significant differences were observed. Additional analyses compared patients with nonsense variants/CNVs to those with missense variants, as well as patients carrying the recurrent c.1129C>T, p.(Arg377Ter) variant to those with other SNVs. No statistically significant differences were identified across any of these comparisons.

#### 3.2.3. Complementary ClinVar and DECIPHER Database Overview

To complement the literature-based cohort, we reviewed publicly submitted *TRAPPC9* variants in ClinVar and DECIPHER. At the time of access, ClinVar reported 1101 germline *TRAPPC9* variants, including 115 pathogenic and 36 likely pathogenic variants; 23 entries were classified as structural variants. Of these, 22 corresponded to large chromosomal duplications encompassing numerous genes on chromosome 8, rather than *TRAPPC9*-specific rearrangements. The remaining entry was an approximately 27.4 kb deletion involving *TRAPPC9* (NC_000008.11:g.(?140417391)(140444832_?)del), reported in the context of a schizophrenia/ASD CNV study rather than as a clinically characterized MRT13 case [39].

DECIPHER reported 22 SNVs, 72 CNVs, and 12 other variant types involving *TRAPPC9*. Among the SNVs, 9 were missense, 9 were predicted loss-of-function SNVs, 3 affected untranslated regions, and 1 was intronic.

## 4. Discussion

### 4.1. Overview

Through a systematic and harmonized analysis of all reported cases, this study provides an updated overview of the clinical and molecular landscape of *TRAPPC9*-related intellectual developmental disorder. The data confirm a recognizable and consistent phenotype, characterized by intellectual disability and brain MRI abnormalities, with microcephaly and motor delay [34]. Neurobehavioral manifestations, including autism spectrum disorder and other behavioral abnormalities, were commonly reported, although showing variable severity across patients. Dysmorphic features were frequently reported but appeared heterogeneous, with no single feature consistently present, indicating the absence of a pathognomonic craniofacial pattern.

From a molecular perspective, most of the reported variants were SNVs or small intragenic deletions/duplications, predominantly predicted to result in loss of function (nonsense and frameshift SNVs), whereas larger CNVs were less common. The complementary ClinVar and DECIPHER overview was consistent with the literature-based findings, supporting the predominance of SNVs and CNVs in the *TRAPPC9* mutational spectrum. Structural variants involving *TRAPPC9* were present in public databases but were mostly represented by large multigenic rearrangements, with very limited evidence for *TRAPPC9*-specific structural events in clinically characterized MRT13 cases.

In this context, the novel case reported here expands the genotypic spectrum of the disorder, highlighting the broader potential of advanced genomic technologies to uncover cryptic structural variants that may remain undetected using conventional approaches.

### 4.2. Novel Structural Mechanism and Diagnostic Implications

The clinical phenotype of our patient closely matched the core features of the disorder, with the exception of the absence of microcephaly, which represents an atypical finding in the context of *TRAPPC9*-related intellectual developmental disorder. Following the identification of the first pathogenic variant, the strong phenotypic concordance supported the hypothesis of a second pathogenic event affecting the other allele. The molecular diagnosis was ultimately established through the identification of a small maternally inherited intragenic deletion in *trans* with the disruption of the paternal allele arising from the breakpoint of an inherited balanced translocation. To our knowledge, this represents the first report of biallelic *TRAPPC9* disruption resulting from the combination of an intragenic deletion and a balanced translocation. No cases involving balanced translocations have been reported so far, highlighting the uniqueness of the molecular mechanism observed in our patient and further expanding the mutational spectrum of the disorder. Notably, both SVs escaped detection by conventional diagnostic approaches, including a conventional 60K array-CGH platform and whole-exome sequencing, highlighting the limitations of standard techniques in identifying cryptic SVs. OGM, by analyzing ultra-high molecular weight DNA molecules, enables high-resolution, genome-wide detection of both balanced and unbalanced structural variants, offering a significant advantage over conventional cytogenetic and sequencing-based methods [40,41]. In this case, it allowed precise breakpoint characterization and directly demonstrated disruption of *TRAPPC9*, thereby uncovering the pathogenic compound heterozygosity.

The clinical history of the proband was complex, including pregnancy complications and neuroradiological findings suggestive of previous hypoxic–ischemic injury. Although these factors may have contributed to selected aspects of the phenotype, the identification of biallelic *TRAPPC9* disruption provides a coherent molecular explanation for the core neurodevelopmental presentation.

These findings underscore the importance of considering structural variants in patients with neurodevelopmental disorders. Although many neurodevelopmental conditions are currently primarily characterized as resulting from single-nucleotide variants (SNVs), the systematic implementation of emerging technologies such as OGM may, in the near future, reveal a substantial proportion of cases arising from complex genomic rearrangement mechanisms that remain undetectable using conventional sequencing approaches. In selected patients with strong clinical suspicion and negative first-tier genetic testing, OGM may therefore improve diagnostic yield by enabling the detection and characterization of cryptic structural alterations, particularly when a balanced translocation requires breakpoint definition and assessment of potential gene disruption.

### 4.3. Genotype–Phenotype Correlations

No statistically significant genotype–phenotype correlations were identified in our cohort. The clinical presentation appeared broadly consistent across patients, supporting the presence of a relatively recognizable and homogeneous neurodevelopmental phenotype associated with *TRAPPC9*-related intellectual developmental disorder. These findings suggest that, despite some variability in individual features, the overall clinical picture is largely independent of the specific type of underlying genetic variant, consistent with a convergent pathogenic mechanism.

### 4.4. Limitations

This study has several limitations that should be considered when interpreting the results. Despite representing the largest harmonized cohort currently available, the total number of patients remains limited due to the ultra-rare nature of *TRAPPC9*-related intellectual developmental disorder. Clinical data were incomplete and variably reported across publications, which may have led to underestimation of certain manifestations. In addition, differences in historical annotation practices and incomplete molecular characterization may still affect the interpretation of the findings despite systematic re-evaluation and re-annotation of all variants. The retrospective design of the literature review and the heterogeneity of previously published reports may also introduce reporting bias. Finally, the review protocol was not prospectively registered, which may limit methodological transparency and reproducibility.

## 5. Conclusions

This study provides an updated and comprehensive overview of the clinical and molecular spectrum of *TRAPPC9*-related intellectual developmental disorder, corroborating the existence of a highly consistent and recognizable core phenotype characterized by intellectual disability, postnatal microcephaly, and neuroimaging abnormalities. Systematic re-evaluation and re-annotation of all reported variants according to the MANE Select reference transcript enabled a more accurate comparison across studies, clarified the distribution of pathogenic variants, and facilitated the identification of recurrent mutational patterns, with a predominance of predicted loss-of-function variants.

The identification of biallelic *TRAPPC9* disruption caused by the combination of a small intragenic deletion and an inherited balanced translocation expands the mutational spectrum of the disorder and emphasizes the importance of considering structural variants in unresolved neurodevelopmental cases. The integration of emerging genomic technologies, such as optical genome mapping, may significantly improve diagnostic yield and should be considered when conventional approaches fail to fully explain the clinical phenotype.

Finally, future studies on larger cohorts will be needed to further refine genotype–phenotype correlations and improve clinical stratification.

## Figures and Tables

**Table 1:** Summary of the clinical features of the 76 patients with *TRAPPC9*-related intellectual developmental disorder.

Feature	n/Total Available	%
Intellectual disability	71/71	100%
Brain MRI abnormalities	47/49	95.9%
White matter abnormalities	40/49	81.6%
Thinning of the corpus callosum	39/49	79.6%
Cerebellar hypoplasia	18/49	36.7%
Reduced cerebral volume	12/49	24.5%
Microcephaly	56/68	82.3%
Motor delay	45/63	71.4%
Dysmorphic features	44/63	69.8%
Obesity	19/36	52.8%
Behavioral abnormalities	32/65	49.2%
Autism spectrum disorder	14/32	43.8%
Sleep disturbances	9/53	17.0%
Epilepsy	11/69	15.9%

**Table 2:** Single Nucleotide Variants (SNVs) identified so far in *TRAPPC9* (NM_001160372).

Protein Variant	cDNA Change	Exon	Patients, n/76
**Nonsense variants**			
p.Arg377Ter	c.1129C>T	7	16
p.Arg472Ter	c.1414C>T	9	8
p.Arg614Ter	c.1840C>T	12	4
p.Glu689Ter	c.2065G>T	14	3
p.Arg974Ter	c.2920C>T	20	3
p.Gln781Ter	c.2341C>T	16	2
p.Glu64Ter	c.190G>T	2	2
p.Gln215Ter	c.643C>T	3	1
p.Arg937Ter	c.2809C>T	19	1
**Frameshift variants**			
p.Leu674TrpfsTer7	c.2021_2024del	14	4
p.Val224CysfsTer13	c.670delG	3	2
p.His708ProfsTer9	c.2121_2122dup	15	2
p.Val666GlyfsTer7	c.1994dup	14	2
p.Trp92ArgfsTer95	c.272_278del	2	2
p.Leu820ValfsTer50	c.2458_2459delCT	17	1
p.Tyr643SerfsTer3	c.1928del	13	1
p.Thr1048ProfsTer8	c.3141delG	22	1
**Splice-site variants**			
p.?	c.730+1G>T	3	4
p.?	c.2557-2A>C	18	2
p.?	c.3055+1G>A	21	2
**Missense variants**			
p.Leu178Pro	c.533T>C	2	2
p.Gly248Glu	c.743G>A	4	2
p.Arg462Cys	c.1384C>T	9	1
p.Pro1026Ser	c.3076C>T	22	1
p.Asn547His	c.1639A>C	11	1
p.Thr15Met	c.44C>T	2	1
p.Arg1042His	c.3125G>A	22	1
p.Arg402Leu	c.1205G>T	8	1
p.Trp456Arg	c.1366T>C	9	1
p.Phe134Leu	c.402C>G	2	1
p.His208Pro	c.623A>C	3	1
p.Gly1071Ser	c.3211G>A	22	1

**Table 3:** Copy Number Variants (CNVs) identified so far in *TRAPPC9*.

CNV	Type
arr[GRCh37] 8q24.3(141297610 × 2,141301047_141332537 × 1,141336595 × 2)	deletion
arr[GRCh37] 8q24.23–q24.3 deletion (~1.7 Mb)	deletion
arr[GRCh37] 8q24.3 (140996534_141185717) × 1	deletion
arr[GRCh37] 8q24.3(141313791_141403956) × 1	deletion
arr[GRCh37] 8q24.3(141460661_141461780) × 0	deletion
[hg18 build] 8q24.3(140879937_141021392) × 0	deletion
exon 12 deletion (WES-CNV analysis)	deletion
arr[GRCh37] 8q24.3(141268759_141364614) × 3	duplication
arr[GRCh37] 8q24.3(141344339_141461062) × 4	duplication

## Data Availability

The data supporting the findings of this study are available within the article and its Supplementary Materials. Additional data are available from the corresponding author upon reasonable request, subject to ethical and privacy restrictions.

## Supplementary Materials

The following supporting information can be downloaded at https://www.mdpi.com/article/10.3390/genes17060658/s1: Table S1: Studies included in the systematic literature review. Figure S1. PRISMA 2020 flow diagram of study selection [42].

## Abbreviations

The following abbreviations are used in this manuscript:

ACMG	American College of Medical Genetics and Genomics
array-CGH	Comparative Genomic Hybridization Array
BWA	Burrows–Wheeler Aligner
CNV/CNVs	Copy Number Variant(s)
DLS	Direct Label and Stain
EDTA	Ethylenediaminetetraacetic Acid
EEG	Electroencephalography
HGVS	Human Genome Variation Society
IGV	Integrative Genomics Viewer
MENA	Middle Eastern and North African
MRI	Magnetic Resonance Imaging
MRT13	Intellectual Developmental Disorder, Autosomal Recessive 13
NF-κB	Nuclear Factor Kappa B
OGM	Optical Genome Mapping
PCR	Polymerase Chain Reaction
PE-WGS	Paired-End Whole-Genome Sequencing
SDS	Standard Deviation Score
SNV/SNVs	Single-Nucleotide Variant(s)
SV/SVs	Structural Variant(s)
TIQ	Total Intelligence Quotient
WES	Whole-Exome Sequencing
