# Supplementary material for: TRAPPC9-Related Intellectual Developmental Disorder: A Systematic Review and a Novel Case of a Complex Structural Variant
Source: Genes (Basel). 2026 Jun 3;17(6):658. doi: 10.3390/genes17060658 (PMC13299287; doi:10.3390/genes17060658)
Supplement: Supplementary file 1 [file genes-17-00658-s001.zip › Supplementary Figure S1. PRISMA 2020 flow diagram of study selection.pdf]

PRISMA 2020 flow diagram for new systematic reviews which included searches of databases, registers and other sources

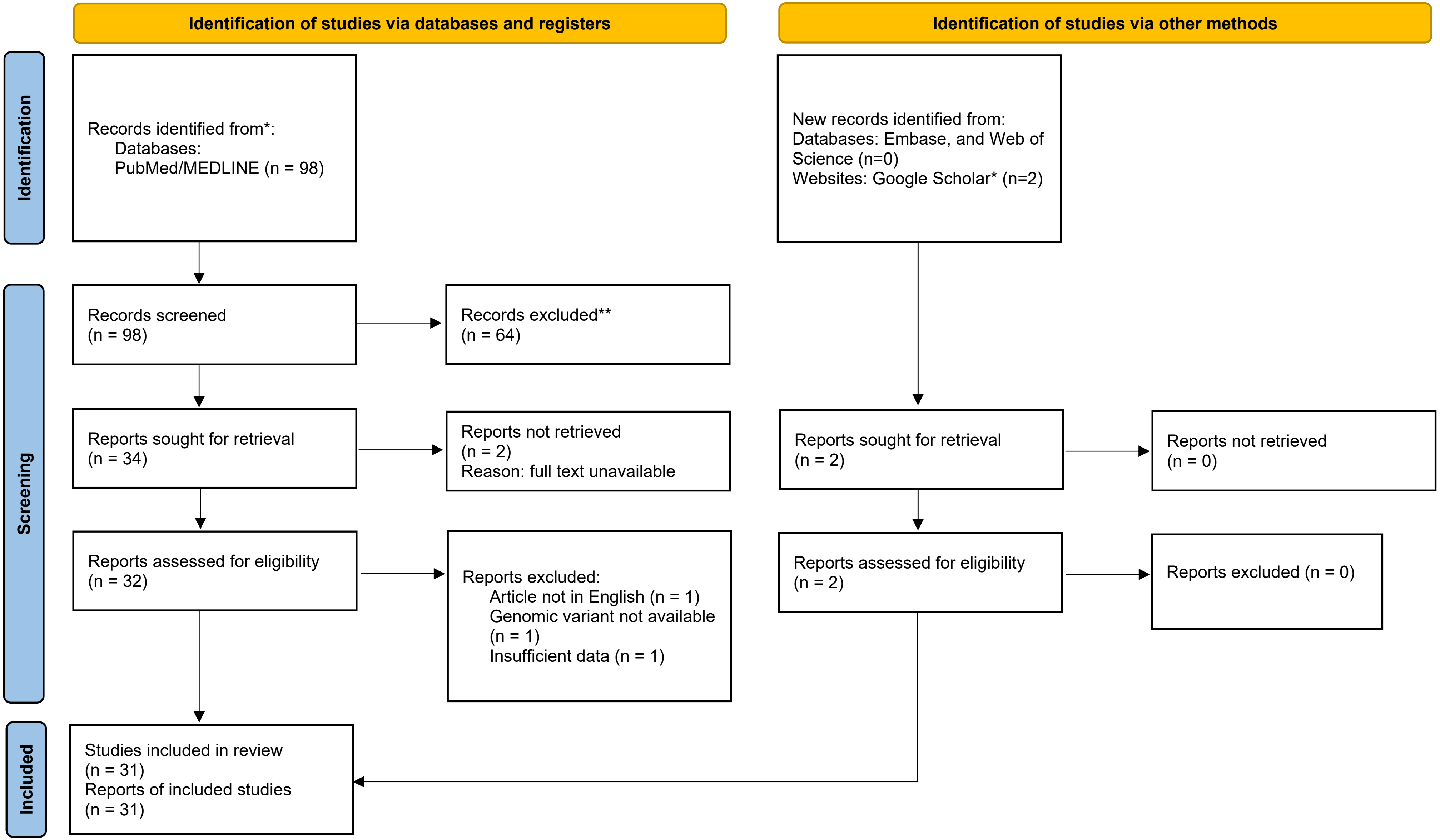

*PubMed/MEDLINE was used as the primary bibliographic database for the literature search. Embase, Web of Science, and Google Scholar were subsequently searched to identify additional records not retrieved through PubMed/MEDLINE. Orphanet, OMIM, and ClinVar were consulted to support disease classification and variant annotation.

** Reasons for exclusion at title/abstract screening included preclinical studies, somatic/oncologic studies, studies on other genes, non-human studies, and studies unrelated to TRAPPC9-related neurodevelopmental disorder.

PRISMA 2020 flow diagram for new systematic reviews which included searches of databases, registers and other sources

Source: Page MJ, et al. BMJ 2021;372:n71. doi: 10.1136/bmj.n71.

This work is licensed under CC BY 4.0. To view a copy of this license, visit https://creativecommons.org/licenses/by/4.0/
